# Recombination Events and Conserved Nature of Receptor Binding Motifs in Coxsackievirus A9 Isolates

**DOI:** 10.3390/v12010068

**Published:** 2020-01-06

**Authors:** Eero Hietanen, Petri Susi

**Affiliations:** Institute of Biomedicine, University of Turku, 20520 Turku, Finland; eevahi@utu.fi

**Keywords:** *Picornaviridae*, coxsackievirus A9, recombination, viral evolution, phylogeny, receptor

## Abstract

Coxsackievirus A9 (CVA9) is an enterically transmitted enterovirus and one of the most pathogenic type among human enteroviruses. CVA9 isolates use a distinctive RGD (Arg-Gly-Asp) motif within VP1 capsid protein that defines its ability to bind to integrin receptor(s) for cellular entry. To investigate CVA9 evolution and pathogenicity, genetic relationships and recombination events were analyzed between 54 novel clinical isolates of CVA9, as well as 21 previously published full length CVA9 sequences from GenBank. Samples were investigated by partial sequencing of the novel VP1 and 3Dpol genes, as well as including the corresponding areas from GenBank sequences. Phylogenetic analyses were combined with clinical data in a further attempt to analyze whether sequence evolution reflects CVA9 pathogenicity in the phylogenies. Furthermore, VP1 gene was also analyzed for receptor binding sites including the RGD motif and the putative heparan sulfate (HS) site. Analysis of the 559-nucleotide-long VP1 sequences identified six clades. Although most of the strains within each clade showed geographical clustering, the grouping pattern of the isolates in the analysis of the VP1 gene was strikingly different from grouping of 3Dpol, which suggests that recombination events may have occurred in the region encoding the nonstructural proteins. Inclusion of clinical data did not provide any evidence of symptom based phylogenetic clustering of CVA9 isolates. Amino acid sequence analysis of the VP1 polypeptide demonstrated that the RGD motif was fully conserved among the isolates while the putative HS binding site was only found in one isolate. These data suggest that integrin binding is essential for virus tropism, but do not explain the symptom repertoire.

## 1. Introduction

Human enteroviruses, small positive-sense, single-stranded RNA viruses in the genus Enterovirus, family Picornaviridae, are subgrouped into four species, Enterovirus A–D, with altogether 116 virus types including polioviruses, coxsackie A and B viruses, echoviruses and numbered enteroviruses. Typing of human enteroviruses is based on genetic distances between VP1 sequences, representing the most heterogenous viral protein. Enteroviral VP1 is the only picornaviral protein linked to serotyping [1,2]. Enteroviruses are common human viruses and endemic in many developing countries. Although most enteroviral infections are subclinical, they cause a spectrum of diseases including mild upper respiratory illness (common cold), febrile rash (hand, foot, and mouth disease and herpangina), aseptic meningitis, pleurodynia, encephalitis, acute flaccid paralysis (paralytic poliomyelitis), and neonatal sepsis-like disease [3,4]. 

Genetic analysis of coxsackievirus A9 (CVA9) revealed that, despite its coxsackie A virus-like pathogenicity in newborn mice, it is genetically more closely related to CV-B viruses than to other CV-As [5,6]. Thus, it is not surprising that CVA9, similarly to other coxsackie B viruses, is one of the most prevalent and pathogenic enteroviruses [3,7,8,9]. The viral genome is translated into a large polyprotein, which typically includes structural proteins (VP1-4) and non-structural proteins (2A-C and 3A-D). In the icosahedral capsid, which mediates virus binding to different cellular receptors [10], VP1, VP2, and VP3 are located on the surface, whereas VP4 faces the internal surface [11,12].

CVA9 VP1 capsid protein carries an unusual C-terminal Arg-Gly-Asp (RGD) tripeptide motif, which is only found in few picornaviruses [10]. Integrin αVβ6 has been shown to be high-affinity receptor in certain cell lines for cellular entry of CVA9 [13,14]. Although important in CVA9 interaction with αV integrins both in vitro and on the cell surface [15,16], there are also studies in which trypsin-treated virus and virus mutants, which lack the RGD motif, are capable of using an RGD-independent pathway in cell entry [15,17,18]. There are also other molecules that have been assigned to CVA9 entry into cells [18,19]. For example, a putative heparan sulfate binding site was recently identified in the VP1 protein [20], but the findings have been disputed in other studies [21].

Enteroviruses, like other RNA viruses, have a high mutation rate due to the lack of proofreading activity during genome replication [22], with an estimate of approximately one mutation being generated per newly synthesized genome [23]. Although random mutations may explain some of the pathogenicity characteristics of enterovirus types, sequence analysis of the VP1 gene has been applied to most molecular epidemiological analysis of enteroviruses including, for example, CVB1 [24], CVB4 [25], CVB5 [26], enterovirus 71 [27], and E30 [28]. Together with the 3Dpol region, which is the furthermost region away from VP1, sequence relationships in other parts of the genome and recombination analyses have been made possible. Recent phylogenetic analysis using different genomic regions (including VP1 and 3Dpol), or complete enterovirus genomes, have provided evidence suggesting that recombination also occurs between other enteroviruses, and that intraspecies recombination is a relatively frequent event in the evolution of enterovirus genomes [29]. Thus, recombination analyses can shed light on the potential movement of enteroviruses between geographical regions, enabling the possibility of analyzing enterovirus epidemiology in more detail. Additional studies regarding aspects such as genetic distances further allow the study of temporal emergence of enteroviruses, adding to the complete epidemiological picture.

In this study, 54 clinical isolates of CVA9, collected from three geographical regions during the last six decades, were subjected to molecular evolutionary analysis. In addition, 21 CVA9 sequences from GenBank were included in the analyses, including the CVA9 prototype strain Griggs. Primarily, the aim of the study was to discover if these viral samples show potential recombination between geographical regions throughout the six-decade-long data set. To examine phylogenetic relationships between the isolates, the VP1 and 3Dpol genome regions were partially sequenced, and phylogenetic trees were constructed using three different methods for building phylogenies. First, maximum likelihood and maximum composite likelihood trees were constructed to infer rudimentary phylogenies. Additionally, a more detailed analysis was carried out in order to construct temporal phylogenies, and thus shed light on the evolution and ancestry of the CVA9 isolates. Furthermore, the secondary aim of the study was to analyze receptor binding sites within VP1 protein. Although RGD has been determined necessary for infection in green monkey kidney (GMK) and A549 cell lines [13,14,30,31,32] and in mouse model [33,34], it is not required for cellular infection of RD and SW480 cell lines [15,16,18]. In contrast, previous studies documenting cell infection regardless of RGD suggest that there are still other undiscovered cellular entry mechanisms for the virus. One such mechanism, suggested as an RGD-independent entry mechanism for the virus, may be heparin binding via a specific T132R/K mutation within the VP1 area. However, more recent blocking studies have demonstrated heparan sulfate (HS) having a more general role in CVA9 infectivity, regardless of the mutation, which could be indicative of the presence of additional heparin binding sites. In this study, an analysis of the RGD and HS binding areas were carried out to examine their prevalence in the samples. Additionally, a HS motif search was carried out against sequence databases in order to find other previously unknown picornaviruses that possess the mutation. Uncovering the details about possible CVA9 recombination would provide information regarding the evolution of these pathogens, as well as their movement across the globe as new epidemics emerge. Together with the presence or lack of receptor binding sites, these data provide us evidence about their roles in virus pathogenicity.

## 2. Materials and Methods 

### 2.1. Clinical Specimens and Symptoms

The novel CVA9 samples used in the study originated from Finland (*n* = 16), the Netherlands (*n* = 30), Denmark (*n* = 1), and Northern America (*n* = 7). Sample details including symptoms are presented in Table 1, which also shows data for the 21 full length CVA9 isolates obtained from GenBank for this study.

### 2.2. Amplification of VP1 and 3Dpol Regions

RNA from CVA9 samples was extracted using a QIAamp MinElute Virus Spin Kit (Qiagen GmbH, Düsseldorf, Germany) following the manufacturer’s instructions. Primers (OS/OAS and IS/IAS) for VP1 and 3Dpol and modified semi-nested PCR protocol were described previously [35]. In short, reverse transcription (RT) step was performed in 10 µL volume and contained 0.5 µL ImProm II (Promega, Madison, WI, USA), 1 µL OAS primer at 1 µM final concentration, ImProm II buffer, RNasin, and ddH_2_O. Samples were incubated at 42 °C for 60 min. Five microliters of RT sample was added to 25 µL PCR1 reaction, which included 0.5 µL Platinum Taq High Fidelity enzyme, 2.5 µL primers at 1 µM final concentration, buffer, and ddH_2_O. Cycling conditions for both VP1 and 3Dpol amplification were 95 °C for 3 min, then 35 cycles of denaturation at 95 °C for 20 s, annealing at 50 °C for 30 s and extension at 72 °C for 60 s. Final extension stage was for 5 min. In snPCR2, 1 µL of PCR1 reaction product was used. 

### 2.3. Nucleotide Sequencing

Amplified DNA from RT-PCR reaction was treated with ExoSAP mixture and directly sequenced using 3730xl DNA Analyzer (Eurofins GATC, Köln, Germany) with the inner sense or antisense primer used for amplification (Eurofins Genomics, Ebersberg, Germany). Overlapping sequences were assembled with Staden Package, trimmed for equal length and aligned with Clustal omega implemented in Seaview program [36], and subjected to evolutionary analyses. All newly generated sequences were deposited in the GenBank database under the accession numbers MN493979-MN494086.

### 2.4. Phylogenetic Analysis

Bootstrapped phylogenetic trees for the VP1 and 3Dpol regions were constructed using the MEGA6 software package. Maximum likelihood (ML) and maximum composite likelihood (MCL) methods were used in the phylogenetic tree construction using the Kimura 2-parameter substitution model [37] with 500 bootstrap replicates. Data used in the analysis included all codon positions, with pairwise deletion for missing data. A Markov Chain Monte Carlo (MCMC) method, implemented in the BEAST software package version 1.8.2 [38], was used for temporal phylogenetic analysis of the data. For the VP1 region, dated sequences were analyzed with a chain length of 60 million, sampling every 6000 states, under the SRD06 substitution model [39] with the assumptions of a relaxed molecular clock model and a constant size coalescent. Other parameters were initially manually checked and adjusted, with final optimization done during the burn-in period. For the 3Dpol region, BEAST analysis was run with a chain length of 90 million to ensure good quality control parameters. Otherwise, the process for the 3Dpol region analysis was carried out the same way as for the VP1 region. Outputs from BEAST for both regions were analyzed within the TRACER v1.6 program to ensure convergence through graphical checks, as well as adequate quality control parameters of posterior distribution (ESS > 200). Final phylogenetic trees from BEAST outputs were constructed with the TreeAnnotator v1.8.2 software to find the maximum clade credibility tree from all the sampled tree states. Finally, the obtained trees were visualized with the FigTree v1.4.2 software.

### 2.5. RGD and HS Binding Site Analysis

To extract the RGD motif from the sample sequences, the raw inner antisense (IAS) and inner sense (IS) files were loaded into Unipro UGENE program [40] where VP1 open reading frames (ORFs) containing the motif were extracted if the sequence data were found to cover the area adequately. These sequences were later translated and trimmed to a 20 aa window around the motif for further analysis with the BioEdit 7.2.5 software [41]. Possible unresolved bases found in some sequences, likely due to RGD motif’s position in the C-terminal end and the Sanger sequencing method used, were manually fixed by using the data from the original sequencing chromatograms. Eventually, a total of 64 sequences were used in the final RGD motif analysis, including the prototype Griggs (GenBank accession number D00627.1) sequence, as well as all available full length CVA9 sequences from GenBank.

The putative HS binding site region was extracted from the originally aligned sequence data with a 29 aa window around the VP1-T132 position as suggested in previous studies [20,21]. The resulting alignment was run through protein BLAST using the non-redundant protein sequence database, excluding CVA9 sequences, with the aim of identifying other potential enteroviruses with present HS binding sites. From the top 250 returned hits, the non-redundant enterovirus sequences were combined with the putative HS region from the full length CVA9 GenBank sequences included in this study and subjected for further analysis.

## 3. Results

### 3.1. Phylogeny of CVA9 VP1 and 3Dpol Regions

A data set of 75 coxsackievirus A9 isolate sequences collected between 1959 and 2016 were analyzed for their VP1 and 3Dpol gene phylogenies. In detail, the complete data set consisted of sequences from three distinct geographical regions: Europe (*n* = 48), Americas (*n* = 11), and Asia-Pacific (*n* = 15), as well as the CVA9 prototype Griggs sequence. Full breakdown of details regarding the isolates is shown in Table 1. 

**Figure 1:**
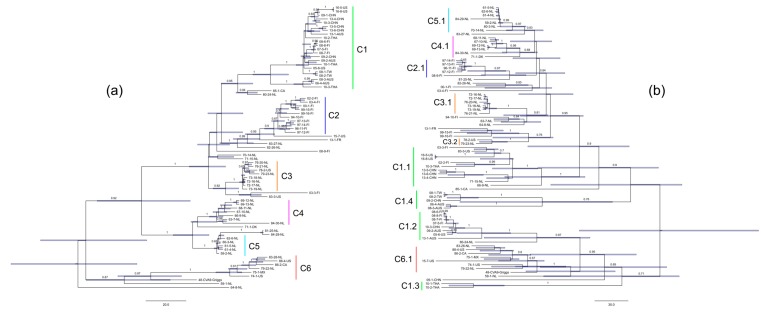
CVA9 phylogenies for (**a**) VP1 and (**b**) 3Dpol regions. Clades for VP1 region were manually assigned based on tree topology, and the 3Dpol region was set to reflect this grouping. Bootstrap values of ≥65% are shown. The light blue bars represent 95% HPDs for ancestor estimates. The scale bar represents time in years.

The temporal phylogenetic analyses done with the BEAST v1.8.2 software package that visualize estimates of CVA9 divergence times are seen in Figure 1. Temporal phylogenies give clues to the evolutionary rate, as well as the time to most recent common ancestor (tMRCA) for the whole data set of VP1 and 3Dpol sequences. The CVA9 VP1 region showed a mean substitution rate of 4.1 × 10^−3^ substitutions/site/year (95% HPD range 3.1–5.0 × 10^−3^) (Table 2). Additionally, VP1 dated the tMRCA back to approximately 1889 (1856–1918). The 3Dpol region showed a mean substitution rate of 3.4 × 10^−3^ substitutions/site/year (95% HPD range 2.3–4.6 × 10^−3^), with the tMRCA dating further back to approximately 1814 (1726–1886) (Table 2). The data may be somewhat biased due to the size of the sample collection and temporal and spatial differences.

The topology of the VP1 phylogeny obtained from BEAST analysis is showing strong clustering by country of origin, and to a degree by collection year, which is similar to what was observed with the initial ML trees. The Asia-Pacific sequences in clade C1 (Figure 1) (06-4-AUS, 08-3-AUS, 09-2-AUS, 13-1-AUS, 09-1-CHN, 09-2-CHN, 10-3-CHN, 13-4-CHN, 13-5-CHN, 13-6-CHN 08-1-TW, 08-2-TW, 10-1-THA, 10-2-THA, 10-3-THA) are seen forming a distinct group with the 2005 and 2016 US (16-5-US, 16-8-US, 05-6-US) and a portion of the 2007–2008 Finnish (07-5-FI, 08-6-FI, 08-7-FI, 08-8-FI) sequences (C1 nucleotide identity between 85–100%, amino acid identity between 97 and 100%). From the European sequences, the remaining Finnish sequences (02-2-FI, 03-4-FI, 00-1-FI, 99-15-FI, 99-16-FI, 94-10-FI, 97-13-FI, 97-14-FI, 96-11-FI, and 97-12-FI) in VP1 otherwise clustered in a single clade C2, with the exception of two sequences (08-9-FI, 03-3-FI) (C2 nucleotide identity between 93 and 98%, amino acid identity between 96 and 100%). Dutch sequences formed three distinct clades C3, C4, and C5. C3 shows Dutch sequences between 1972–1979 (76-20-NL, 76-21-NL, 79-23-NL, 73-18-NL, 72-16-NL, 72-17-NL, 73-19-NL), with a single US sequence from the late 1970s (78-2-US) (C3 nucleotide identity between 95 and 100%, amino acid identity between 98 and 100%). C4 shows sequences primarily between 1963 and 1969 (69-12-NL, 69-13-NL, 68-11-NL, 67-10-NL, 66-9-NL, 63-7-NL), with a single outlier of a Dutch sequence from 1984 (84-30-NL) (C4 nucleotide identity between 92 and 99%, amino acid identity between 95 and 99%). C5 shows Dutch sequences between 1959 and 1962 (62-6-NL, 60-3-NL, 61-5-NL, 61-4-NL, 59-2-NL) (C5 nucleotide identity between 98 and 99%, amino acid identity between 99 and 100%). Dutch sequences from the 1980s were seen generally more scattered around the VP1 tree and not clustering together. Instead, they positioned close to C1 (80-24-NL), C2 (83-27-NL, 82-26-NL), C3 (83-28-NL), C5 (81-25-NL, 84-29-NL), and within C6 (83-28-NL). The American sequences formed one distinct clade C6 that contained all of the sequences before the year 2000 (88-4-US, 82-2-CA, 75-1-MX, 74-1-US), with the exception of two sequences (85-1-CA, 78-2-US) (C6 nucleotide identity between 85 and 95%, amino acid identity between 98–100%). Interestingly, all of the American sequences collected later, between 2005 and 2016 are not seen clustering in any close proximity of the older American sequences. From these more recent American sequences 16-5-US, 16-8-US, and 05-6-US are seen included in C1 with Asia-Pacific and Finnish sequences, while 15-7-US positions close to C2 where majority of Finnish sequences are clustered.

On the side of the 3Dpol phylogeny, the tree topology is immediately more disorganized compared to the VP1 topology (Figure 1). While some clusters are still seen forming by geographic area, the clusters are noticeably smaller compared to VP1. Additionally, there are a higher number of individual sequences not positioning in distinct clades, with noticeably higher tMRCA estimates. Clade C1 from the VP1 phylogeny that contained Asia-Pacific, Finnish 2007–2008, and a single 2005 US sequence, is seen breaking up into four smaller clades (C1.1, C1.2, C1.3, C1.4) in the 3Dpol phylogeny. Clade C1.1 contains the Chinese sequences from 2013 (13-4-CHN, 13-5-CHN, 13-6-CHN), the 2016 US sequences (16-5-US, 16-8-US), and one 2010 Thai sequence (10-3-THA). On the other hand, the clade contains some Finnish (02-2-FI, 03-3-FI) and Dutch (71-15-NL) sequences that were not seen in the VP1 phylogeny. Clade C1.2 contains the 2007–2008 Finnish, 2010 Chinese, 2005 US, and 2009 and 2013 Australian sequences. An exception in C1.2 is that one of the Finnish sequences (08-9-FI) has positioned near other Finnish sequences in C2.1. C1.3 contains just two Thai sequences from 2010 (10-1-THA, 10-2-THA). Although C1.3 shares a bigger clade with 09-1-CHN sequences, the time to the coalescent is seen increasing substantially. C1.4 is seen containing both of the Taiwanese sequences (08-1-TW, 08-2-TW) clustered together with the remaining Chinese sequence (09-2-CHN). Additionally, C1.4 contains the remaining Australian sequences (06-4-AUS, 08-3-AUS), albeit with again noticeably higher tMRCA estimate compared to VP1.

From the European isolates, clade C2 in VP1 contained majority of the Finnish sequences, collected between 1994 and 2003. In the 3Dpol phylogeny, C2 lost six of its members and only forms a smaller cluster of four sequences seen in C2.1 (97-12-FI, 96-11-FI, 97-14-FI, 97-13-FI). The other six isolates from C2 (00-1-FI, 03-4-FI, 94-10-FI, 99-15-FI, 99-16-FI, 02-2-FI) neither form any coherent cluster together on the 3Dpol side, nor do they cluster together with the 2007–2008 Finnish sequences. Clade C3 is seen breaking in to two clades C3.1 and C3.2 in the 3Dpol phylogeny. C3.1 contains majority of the C3 sequences (78-18-NL, 73-19-NL, 76-20-NL, 72-17-NL, 72-16-NL, and 76-21-NL) and C3.2 is seen positioning further away including the two remaining sequences (78-2-US and 79-23-NL). Clade C4 of Dutch sequences was largely intact in the 3Dpol phylogeny, as seen in clade C4.1. Two exceptions were seen with 63-7-NL positioning closer to C3.1, and 66-9-NL positioning closer C1.1. C5, the last clade from VP1 that contained primarily Dutch isolates, was found intact in the 3Dpol phylogeny as seen in clade 5.1. The Dutch sequences from the 1980s that were already seen dispersing around the tree on the VP1 side, are similarly seen dispersing around different clades in the 3Dpol phylogenetic tree, with no clear clustering by collection year seen, contrary to most of the other isolates.

American isolates were previously seen in the VP1 phylogeny forming the clade C6, as well as positioning in the Asia-Pacific clade C1, with C6 containing US, Mexico, and Canadian sequences from the 1970s and 1980s, whereas C1 contained more recent US sequences from 2005 and 2016. Clade C6 was found largely intact in the 3Dpol phylogeny with two noticeable changes. First, the 2015 US sequence 15-7-US, that did not cluster with other American sequences in VP1, is now seen part of C6 on the 3Dpol side. Secondly, C6 lost the member 83-3-US, which positioned closer to C1.1 in the 3Dpol phylogeny.

### 3.2. Receptor Binding Site Analysis

**Figure 2:**
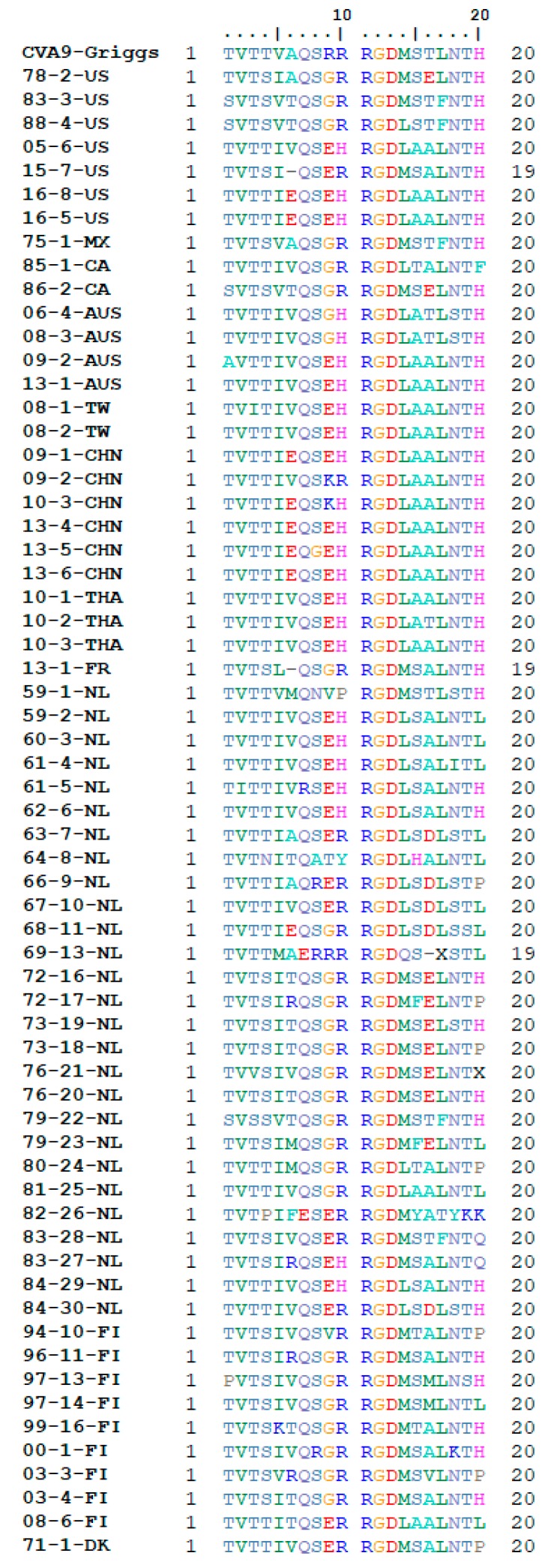
CVA9 RGD binding site analysis showing fully conserved nature of the RGD motif in all of the analyzed isolates. VP1 position 290 in the prototype strain Griggs is equivalent to position 11 in the alignment.

Previous studies have shown that the arginine-glycine-aspartic acid (RGD) motif found in the VP1 capsid protein of CVA9 has a role in cell entry. This motif mediates cell entry also in other picornaviruses such as echovirus 9 (Barty strain), human parechoviruses 1, 2, 4, 5, 6, and foot-and-mouth disease viruses, by binding to several different previously identified integrins [42]. In the case of CVA9, the RGD motif has been found to recognize and bind to the αVβ3 and αVβ6 integrins. Analysis of the 64 CVA9 isolates found that all of them possessed the RGD motif (Figure 2). Several different mutations around the RGD motif were seen. L/M mutations at position RGD + 1 and L/F mutations at position RGD + 4.

**Figure 3:**
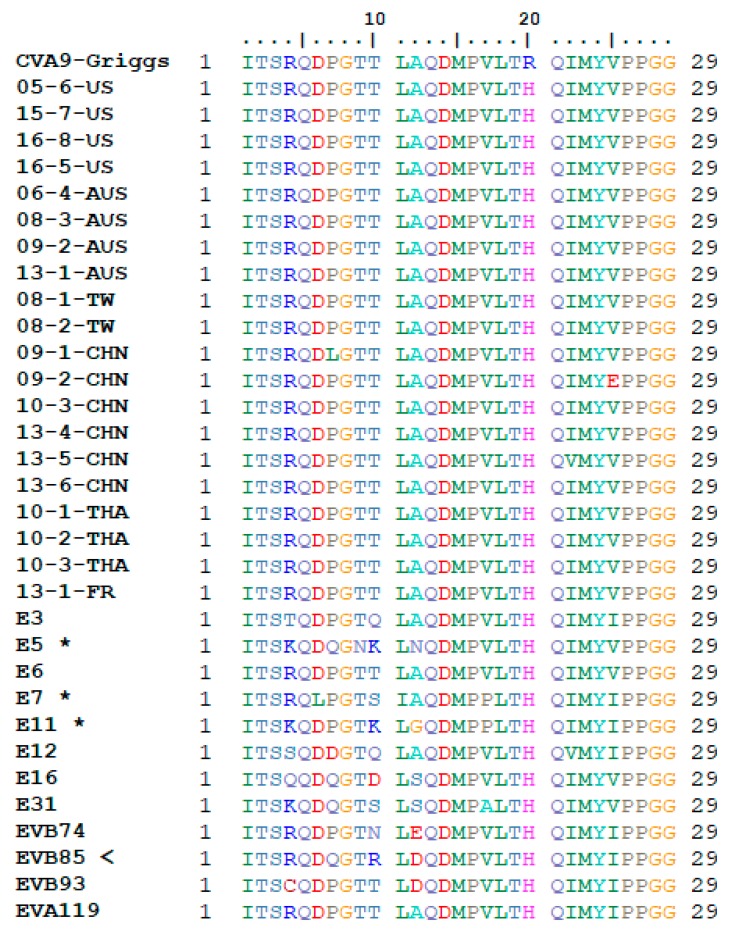
CVA9 HS binding domain. VP1 position 132 in the prototype strain Griggs is equivalent to position 10 in the alignment, where the T132R/K mutation has been identified for enabling HS binding. Three types with previously detected HS binding sites are marked with an asterisk, while the EVB85 marked with an arrow is new. None of the full length CVA9 sequences from GenBank exhibited the T132R/K mutation.

BLAST search and analysis of the putative heparin binding site, described in a previous study [21], resulted in twelve non-redundant enterovirus types that had the proposed motif (E3, E5, E6, E7, E11, E12, E16, E31, EVB74, EVB85, EVB93, and EVA119). The motif for the putative heparin binding site for the viruses obtained through BLAST, as well as the full length CVA9 sequences obtained from GenBank for this study can be seen in Figure 3. Results for the heparin binding site analysis for the other novel CVA9 isolates used in this study have been previously published by others [21]. From the BLAST results, three of the twelve enterovirus types (E5, E7, and E11) were already previously suggested to possess the heparin binding motif and the relevant T132R/K mutation, whereas the nine remaining species are new to our knowledge [20]. However, the specific E7 isolate obtained through BLAST did not have this mutation present. The T132R/K mutation suggested for being responsible for enabling heparin binding was found in one of the nine newly proposed enterovirus types, EVB85 with a T132R mutation. Five out of the eight remaining types showed T132/Q/S/D/N mutations at this position (E3, E12, E16, E31, and EVB74). None of the full length CVA9 sequences obtained from GenBank exhibited the T132R/K mutation thought to enable heparin binding.

## 4. Discussion

Virus epidemiological studies have proven to be valuable in understanding the nature of virus evolution and clinical features. For example, in the case of enteroviruses, extensive epidemiological works, such as virus identification and typing, have been done with the poliovirus during eradication process. Furthermore, in the modern world population mobility is at a level where pathogens can travel across the planet within a day. This high mobility of people has highlighted further need for understanding how pathogens have moved geographically and evolved along the way, which in turn has a relationship with epidemiological research [43]. 

In this study, 75 clinical isolates of coxsackievirus A9 (CVA9) from three geographical regions, collected between 1959 and 2016, were subjected to phylogenetic analysis in order to study the occurrence of recombination and possible links to CVA9 pathogenicity. Additionally, genomic analysis of the RGD and HS receptor binding sites was carried out to shed light on possible mutations that might affect tropism and pathogenicity. Enteroviruses are among the most common disease-causing viruses in humans, and coxsackieviruses belonging to this family are a cause of wide range of diseases in humans, such as meningitis, myocarditis, respiratory, and gastrointestinal infections. Thus, understanding possible recombination events between isolates originating from different geographical areas will be important in understanding how the species has evolved and what effects it might have on epidemiology. Furthermore, closer analysis of receptor binding areas gives valuable information regarding virus cell entry mechanisms and tropism. Combining available clinical data with the phylogenetic and sequence level analyses will further allow us to speculate on the possible effects that CVA9 evolution has on pathogenicity.

Evidence of possible recombination events was gathered by building phylogenetic trees of the VP1 and 3Dpol genomic areas of all 75 CVA9 isolates. Within the VP1 area phylogenetic tree, six bootstrap supported clades were identified, which resulted in the isolates grouping largely by country of origin and collection year. The clades identified within VP1 were compared to the phylogenetic tree of the 3Dpol area, which showed evidence of incongruence in the tree topologies (Figure 1). This incongruence suggests possible recombination between CVA9 isolates in clade C1 and C6. Clade C1 contained sequences gathered from Finland in 2007–2008, Asia-Pacific region between 2006 and 2013, and a single US sequence from 2005. On the other hand, C6 contained isolates mainly from the Americas gathered between 1974 and 1988, as well as a Dutch sequence from 1979. Isolates from C1 were seen splitting into four smaller clades in the 3Dpol phylogeny, with C1.2 and C1.3 showing closer relationship to C6.1. Furthermore, Finnish CVA9 sequences were seen dispersing to a noticeable degree in the 3Dpol phylogeny compared to VP1, with isolates from C2 dispersing on the 3Dpol side and showing incongruent positioning compared to their VP1 counterparts. Additionally, Dutch isolates from C3 were seen mixing with the Finnish sequences from C2, as is seen on the 3Dpol phylogeny with the breaking of C3 into C3.1 and C3.2 while exhibiting closer relationships with Finnish sequences 94-10-FI, 99-15-FI, and 99-16-FI. Furthermore, Dutch sequences from C4 were also seen positioning significantly closer to Finnish sequences from C2 in the 3Dpol phylogeny, as is evident by the positions of clades C4.1 and C2.1. Overall, the phylogenetic analysis shows signs of continuous recombination between the CVA9 isolates. The tMRCA results show a rather distant ancestor for the viruses, but this could be explained due to the nature of the data set. Importantly, it should be noted that tMRCA estimates with these data are extrapolatory in nature, as the temporal analyses were done without any old reference sequence available to calibrate the data further. Thus, conclusions based on the tMRCA estimate of the basal node of the phylogenetic tree should be done with care in mind. The involvement of sequence data from additional countries, and spanning several decades from each country, would give a more detailed overview of the evolution of these viruses.

Combining clinical data with phylogenetic analyses gives an opportunity to speculate on what possible effect sequence evolution might have on CVA9 pathogenicity, and if this effect would be extracted from the data. VP1 and 3Dpol phylogenies were generated to visualize any discernible correlations between the tree topologies of the isolates and clinical data. The data set contained 21 isolates from patients with symptoms of meningitis (08-6-FI, 08-7-FI, 08-8-FI, 08-9-FI, 09-1-CHN, 09-2-CHN, 60-3-NL, 61-4-NL, 61-5-NL, 70-14-NL, 71-15-NL, 72-16-NL, 73-18-NL, 73-19-NL, 79-22-NL, 83-3-US, 85-1-CA, 96-11-FI, 97-12-FI, 97-13-FI, and 97-14-FI). The majority of these isolates are positioned in clades C1, C2, C3, C5, and C6 in the VP1 phylogeny. Additionally, isolates 08-9-FI, 85-1-CA, 70-14-NL, and 71-15-NL are not part of any previously defined clade. Comparing this topology to the positioning of the isolates in the 3Dpol phylogeny, there is no clear shift in the placement of meningitis causing CVA9 isolates. The isolates are seen grouping similarly as on the VP1. That is, the meningitis causing isolates within the individual clades in the VP1 phylogeny form similar clusters in the 3Dpol phylogeny. Furthermore, there is no perceived effect of these clusters pooling together or closer to each other in any significant manner in either phylogeny based on symptoms, but rather the grouping is seen to be more related to the country of origin and collection dates as previously stated. In a similar fashion, no conclusions can be made on pathogenicity based on the phylogenies by looking at cases where the clinical isolates caused diarrhea in patients. A more complete record of clinical data for symptoms, as well as more longitudinal data, would to highlight evolutionary effects and could help in analyzing these possible effects.

Regarding the RGD motif, the leucine flanking RGD at position +1 has been previously found to stabilize integrin binding to the α5β1 and αVβ3 receptors in another picornavirus, the foot-and-mouth disease virus (FMDV) [44]. Additional studies have also shown the leucines at RGD + 1 and +4 to be important for stable binding to integrin αVβ6 [45]. The RGD surrounding area is shown to be highly variable with multiple mutations visible on both the *n*- and C-terminal side of the motif. However, the exact effect of these mutations on CVA9 infection remains unknown. At the same time the highly conserved nature of the RGD motif is indicative of its likely role in the early steps of multicellular infection since αV receptors are primarily expressed in epithelial cells.

The heparin binding has been suggested to occur via a T–R/K mutation in the position 132 (T132R/K) of VP1 in CVA9. The T132R/K mutation was proposed to cluster positive charges around the 5-fold axis, thus allowing binding to heparan sulfate on the cell surface [20]. The T132R/K mutation identified in three other enterovirus types suggests that these types may use HS as a cell entry receptor. However, previous studies have shown that blocking of this site does not necessarily inhibit infectivity [21]. Thus, it can be assumed there being additional putative binding sites that warrant further study in order to gain a comprehensive view of the role of HS in receptor-mediated infection by CVA9. Additionally, BLAST analyses exploring this possibility showed three other previously known enteroviruses (E5, E7, and E11) and one previously unknown (EVB85) also possess the T132R/K mutation thought to be responsible for enabling HS binding. The five enterovirus types (E3, E12, E16, E31, and EVB74) discovered possessing a mutation other than T132R/K at this point included the mutations T132/Q/S/D/N. None of these amino acids possess a positive charge, with glutamine, serine, and asparagine having a neutral charge, and aspartic acid having a negative charge. Furthermore, studies have shown that heparan sulfate binding motifs are composed of sequences of the type XBBXBX and XBBBXXBX, where B is a basic residue, and X is somewhat neutral or hydrophobic residue [46]. None of the found Q/S/D/N mutations fit into the suggested model of the motif structure. However, ultimately their effect on CVA9 infection remains unexplored.

The RGD and HS binding motifs are both located in the VP1 gene, which encodes for one of the four capsid proteins and is a highly variable area of the genome. Furthermore, the VP1 area is used in virus typing and as such is an area of great interest when studying viral pathogenicity. Thus, any significant changes in these motifs would be of high interest if detected. In this data set, we show that RGD remains to exhibit conserved nature in clinical samples. The RGD motif was found to be fully conserved in all of the isolates, and while some mutations surrounding the immediate motif were found, the motif itself remained unchanged, both highlighting its importance in infection, but also diminishing its importance in changes of tropism. The putative heparin binding motif was found to be present in some enterovirus types, and the site has been proposed to play a role in enterovirus infection [20]. However, the T132R/K mutation thought to enable heparin binding is found to be very rare in these data, with none of the GenBank CVA9 sequences possessing the mutation, and only 10 out of 54 of the novel CVA9 sequences possessing a T132R/K mutation as published previously [21]. Consequently, any correlations cannot be speculated between changes in CVA9 pathogenicity and the changes, or lack thereof, of the RGD and HS binding motifs. Thus, changes seen in CVA9 pathogenicity must originate from elsewhere in the sequence.

## 5. Conclusions

In conclusion, the analysis presented in this study suggests that CVA9 exhibits continuous recombination, taking place over multiple decades as well as across vast geographical areas. The data set used in this study showed possible recombination events taking place by analyzing incongruences observed in the topologies of the phylogenetic trees. A clade containing CVA9 isolates from the Asia-Pacific region collected between 2006 and 2013, US isolates collected in 2005 and 2016, and Finnish isolates collected in the 2000s exhibited possible recombination with sequences collected from the US between 1970 and 1980. Dutch sequences collected between 1972 and 1979 and present in the clade C3 showed possible recombination with Finnish sequences from 1990s, which were within the clade C2 in VP1, and with some Dutch sequences from the early 1960s. In addition to phylogenetic recombination analyses, the clinical data was analyzed to visualize if the changes in tree topologies reflect changes in CVA9 pathogenicity. Based on the analyses of meningitis and diarrhea clinical data, no conclusions can be made from these data as there were no discernible changes in the phylogenies that would reflect the clinical symptoms. A more complete record of both clinical and longitudinal data is required to more accurately reflect sequence evolution and to make more educated conclusions on these connections. Furthermore, analysis of the RGD and HS receptor binding sites revealed that RGD remains conserved while the role of HS site is elusive. The RGD motifs highly conserved state in nature warrants further studies of its function in multicellular infection, as it has been shown not to be required for infection in certain cell lines [15,16,18]. Additionally, the observed mutations targeting leucines at positions RGD + 1 and +4 around the motif could have an effect on binding affinity. Although the highly conserved nature of RGD highlights its importance in infection, it does not bring forward any new information regarding CVA9 pathogenicity. Thus, any differences between pathogenicity of CVA9 isolates must be explained by sequence evolution outside RGD region. The putative heparin binding site, thought to be enabled by the VP1-T132R/K mutation, was found to be rare. The T–R mutation was found in only one of the 54 novel CVA9 isolates within this data set, whereas the T–K mutation was found in nine, as described in a previous study [21]. Additionally, the putative HS binding site was not found present in any of the full length CVA9 sequences gathered from GenBank for this study. BLAST analyses found that other enteroviruses could also possess the HS binding mutation, suggesting that echoviruses 5, 7, and 11 also have the motif in question, all of which were already described by a previous study [20]. However, previous inhibition studies have shown that CVA9 may possess additional unidentified HS binding sites within its sequence [21], making these sites a good target for further studies in order gain a more complete picture of the infection mechanisms of CVA9, as well as other enterovirus types.

## Figures and Tables

**Table 1:** CVA9 sample isolation years, countries, sample types, symptoms, and accession numbers.

Sample ID ^1^	Isolation Year	Country of Origin	Sample Type	Clinical Symptoms	GenBank Accession ^2^
06-4-AUS	2008	Australia	Feces	N/A	MF678346.1
08-3-AUS	2008	Australia	Feces	N/A	MF678309.1
09-2-AUS	2009	Australia	Feces	N/A	MF678303.1
13-1-AUS	2013	Australia	Feces	N/A	MF678330.1
85-1-CA	1985	Canada	Feces	Meningitis	MN494025/MN494079
86-2-CA	1986	Canada	Feces	Headache, diarrhea, URI	MN494026/MN494080
09-1-CHN	2009	China	Feces	Meningitis	KM890277.1
09-2-CHN	2009	China	Feces	Meningitis	KM890278.1
10-3-CHN	2010	China	N/A	N/A	KP266574.1
13-4-CHN	2013	China	CSF	HFMD	KP289437.1
13-5-CHN	2013	China	CSF	HFMD	KP290111.1
13-6-CHN	2013	China	CSF	HFMD	KP289434.1
71-1-DK	1971	Denmark	Feces	Fever	MN494022/MN494076
00-1-FI	2000	Finland	Feces	Gastroenteritis	MN493979/MN494033
02-2-FI	2002	Finland	Feces	Diarrhea	MN493980/MN494034
03-3-FI	2003	Finland	Feces	Diarrhea	MN493981/MN494035
03-4-FI	2003	Finland	Feces	Diarrhea	MN493982/MN494036
07-5-FI	2007	Finland	Feces	Diarrhea	MN493983/MN494037
08-6-FI	2008	Finland	CSF	Meningitis	MN493984/MN494038
08-7-FI	2008	Finland	CSF	Meningitis	MN493985/MN494039
08-8-FI	2008	Finland	CSF	Meningitis	MN493986/MN494040
08-9-FI	2008	Finland	CSF	Meningitis	MN493987/MN494041
94-10-FI	1994	Finland	Feces	Cerebellitis	MN494030/MN494084
96-11-FI	1996	Finland	Feces	Meningitis	MN494032/MN494086
97-12-FI	1997	Finland	Throat swab	Meningitis	MN494018/MN494072
97-13-FI	1997	Finland	CSF	Meningitis	MN494019/MN494073
97-14-FI	1997	Finland	CSF	Meningitis	MN494031/MN494085
99-15-FI	1999	Finland	Nasal swab	Exanthema	MN494020/MN494074
99-16-FI	1999	Finland	Nasal swab	Exanthema	MN494021/MN494075
13-1-FR	2013	France	N/A	N/A	KM201659.1
75-1-MX	1975	Mexico	Feces	Diarrhea	MN494027/MN494081
59-1-NL	1959	Netherlands	Feces	Diarrhea, exanthema	MN493988/MN494042
59-2-NL	1959	Netherlands	Feces	Exanthema, leucopenia	MN493989/MN494043
60-3-NL	1960	Netherlands	CSF	Meningitis	MN493990/MN494044
61-4-NL	1961	Netherlands	CSF	Meningitis	MN493991/MN494045
61-5-NL	1961	Netherlands	CSF	Meningitis	MN493992/MN494046
62-6-NL	1962	Netherlands	Feces	Fever, vomiting	MN493993/MN494047
63-7-NL	1963	Netherlands	Feces	Fever, vomiting	MN493994/MN494048
64-8-NL	1964	Netherlands	Feces	Fever, vomiting	MN493995/MN494049
66-9-NL	1966	Netherlands	Feces	Gastroenteritis	MN493996/MN494050
67-10-NL	1967	Netherlands	Feces	Myelitis, fever	MN493997/MN494051
68-11-NL	1968	Netherlands	Feces	Diarrhea	MN493998/MN494052
69-12-NL	1969	Netherlands	Throat swab	Convulsion	MN493999/MN494053
69-13-NL	1969	Netherlands	CSF	Headache	MN494000/MN494054
70-14-NL	1970	Netherlands	CSF	Meningitis	MN494001/MN494055
71-15-NL	1971	Netherlands	Feces	Meningitis	MN494002/MN494056
72-16-NL	1972	Netherlands	Feces	Meningitis	MN494003/MN494057
72-17-NL	1972	Netherlands	Feces	Facial paralysis	MN494004/MN494058
73-18-NL	1973	Netherlands	Feces	Meningitis	MN494005/MN494059
73-19-NL	1973	Netherlands	Feces	Meningitis	MN494006/MN494060
76-20-NL	1976	Netherlands	Throat swab	Gastroenteritis	MN494007/MN494061
76-21-NL	1976	Netherlands	Feces	Thrombocytopenia	MN494008/MN494062
79-22-NL	1979	Netherlands	Throat swab	Meningitis	MN494009/MN494063
79-23-NL	1979	Netherlands	Feces	Pneumonia	MN494010/MN494064
80-24-NL	1980	Netherlands	Throat swab	Encephalitis	MN494011/MN494065
81-25-NL	1981	Netherlands	Feces	Respiratory symptoms	MN494012/MN494066
82-26-NL	1982	Netherlands	Feces	Diarrhea	MN494013/MN494067
83-27-NL	1983	Netherlands	Feces	Gastroenteritis	MN494014/MN494068
83-28-NL	1983	Netherlands	Feces	N/A	MN494015/MN494069
84-29-NL	1984	Netherlands	urine	Dyspnoea	MN494016/MN494070
84-30-NL	1984	Netherlands	Feces	N/A	MN494017/MN494071
08-1-TW	2008	Taiwan	N/A	N/A	KT353721.1
08-2-TW	2008	Taiwan	N/A	N/A	MF422557.1
10-1-THA	2010	Thailand	Throat/nasal swab	N/A	KU574636.1
10-2-THA	2010	Thailand	Nasal swab	Influenza-like illness	KU574637.1
10-3-THA	2010	Thailand	Nasal swab	Influenza-like illness	KU574638.1
05-6-US	2005	USA	N/A	N/A	MH752987.1
15-7-US	2015	USA	Feces	N/A	MN166093.1
16-5-US	2016	USA	N/A	N/A	KY674974.1
16-8-US	2016	USA	N/A	N/A	KY674976.1
74-1-US	1974	USA	Feces	Headache	MN494024/MN494078
78-2-US	1978	USA	Feces	Diarrhea	MN494029/MN494083
83-3-US	1983	USA	Feces	Meningitis	MN494023/MN494077
88-4-US	1988	USA	Feces	Headache	MN494028/MN494082
CVA9-Griggs	1948	USA	N/A	N/A	D00627.1

^1^ Sample IDs listed as [year]-[sequence number]-[country code]. ^2^ Accession numbers listed as [VP1 acc.]/[3Dpol acc.] for partial sequences.

**Table 2:** Markov Chain Monte Carlo (MCMC) BEAST analysis statistics for CVA9 VP1 and 3Dpol regions.

tMRCA (year) ^1^		Substitution Rate (10^−3^ subs./site/year) ^2^	
VP1	3Dpol	VP1	3Dpol
1889 (1856–1918)	1814 (1726–1886)	4.1 (3.1–5.0)	3.4 (2.3–4.6)

^1^ Estimated year of tMRCA (95% CI in parentheses). ^2^ Estimated mean substitution rates (95% HPD range in parentheses).
